# The role of sleep disorders in the motoric and cognitive trajectories of older physically frail sarcopenic and healthy active subjects: SOMNUS-DARE, a prospective cohort study

**DOI:** 10.1186/s12877-026-07275-3

**Published:** 2026-03-18

**Authors:** Marco Salvi, Irene Zucchini, Fulvio Lauretani, Beatrice Tanzi, Crescenzo Testa, Giorgio Ughetti, Liborio Parrino, Carlotta Mutti, Francesco Rausa, Irene Pollara, Margherita Soglia, Nicola Sverzellati, Francesca Bozzetti, Livia Ruffini, Caterina Ghetti, Rosalia Aloe, Giampaolo Niccoli, Caterina Caminiti, Matteo Puntoni, Giuseppe Maglietta, Marcello Maggio, SOMNUS-DARE Investigators, Laura Torlai Triglia, Andrea Stefanizzi, Filippo Luca Gurgoglione, Chiara Cattabiani, Sabrina Spaggiari, Patrizia Cagna, Gertila Rrapaj, Valeria Ribattezzato, Elisa Galli, Marta Leone, Elisabeth Imperatore, Salvatore Vincenzo Anfuso, Silvia Lazzarelli, Denisa Balan

**Affiliations:** 1 Geriatric Clinic Unit, University Hospital of Parma, Parma, Italy; 2 Department of Medicine and Surgery, University of Parma, Parma, Italy; 3 Interdepartmental Centre for Sleep Medicine, University of Parma, Parma, Italy; 4 Sleep Disorders Center, University Hospital of Parma, Parma, Italy; 5 Radiological Sciences Unit, University Hospital of Parma, Parma, Italy; 6 Neuroradiology Unit, University Hospital of Parma, Parma, Italy; 7 Nuclear Medicine Division, University Hospital of Parma, Parma, Italy; 8 Medical Physics Unit, University Hospital of Parma, Parma, Italy; 9 Laboratory of Clinical Chemistry and Haematology, University Hospital of Parma, Parma, Italy; 10 Division of Cardiology, University Hospital of Parma, Parma, Italy; 11 Clinical and Epidemiological Research Unit, University Hospital of Parma, Parma, Italy

**Keywords:** Digital health, Sleep disorders, Ageing trajectories, Multidisciplinary approach

## Abstract

**Background:**

Sarcopenia, altered bone and systemic metabolism, physical frailty and cognitive decline are age-dependent and interrelated phenomena. Sleep disorders which are erroneously considered as part of the aging process might be the unifying underlying mechanism of accelerated aging. It is well-known that impaired sleep health could predict neurodegeneration and functional decline. However, in current clinical practice, sleep disorders are not routinely screened and only a minority of cases require second-tier specialist techniques. The delay of their assessment, without a comprehensive evaluation, results in higher healthcare costs. The aim of SOMNUS-DARE project is to detect, in a translational manner, the role of sleep disorders in the genesis of physical frailty, sarcopenia, mild cognitive impairment, and to measure their contribution to the progression of these phenomena towards mobility-disability and dementia.

**Methods:**

SOMNUS-DARE project will evaluate a sample of 300 community-dwelling healthy active and physically frail sarcopenic subjects, aged 65 years or older, free of cognitive impairment and self-sufficient in basic activities of daily living. The effect of sleep disorders on physical, cognitive and cardiovascular function, systemic, bone and muscle metabolism and body composition will be prospectively assessed by performing: comprehensive geriatric assessment; sleep analysis and environmental monitoring; brain MRI, amyloid positron emission tomography; bone densitometry; radiofrequency echographic multi spectrometry; muscle ultrasound; bioimpedance analysis; cardiac ultrasound; dynamic blood pressure monitoring; blood and saliva measurements.

**Discussion:**

The project will make it possible to analyse an extremely relevant public health problem which is still underestimated in the current clinical practice. The combination of traditional diagnostics and innovative digital health techniques, will allow a translational and multidimensional analysis of sleep disorders and to weigh their impact on physical and cognitive decline and quality-of-life trajectories. The expected results will highlight the importance of sleep as a fundamental determinant of health and will contribute to the activation of clinical diagnostic and therapeutic pathways in older individuals where the tailored approach might contribute to substantial benefit.

**Trial registration:**

Clinical Trial Number: NCT06944600; Clinical Trial Registry: ClinicalTrials.gov; Registration Date: April 17th, 2025.

## Background

### The complex interplay between sleep health, brain, muscle, bone, body composition, endocrine and cardiovascular systems

**Fig. 1:**
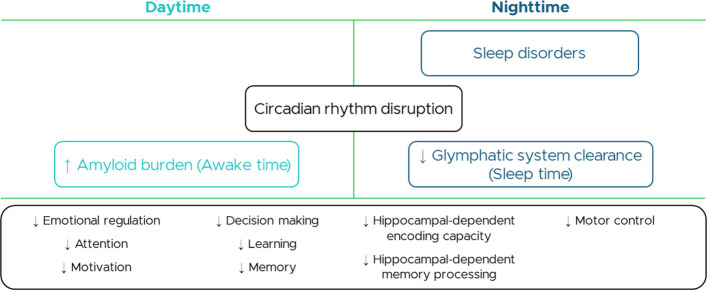
Schematic representation of the glymphatic theory

Skeletal muscle health and brain integrity are strictly interconnected, with sleep as a shared regulatory factor for their trajectories. This contribution of sleep profile could be particularly relevant for sarcopenia, physical frailty, and mild cognitive impairment (MCI) in older adults. In this population, sleep disorders—often misattributed to normal aging—can precipitate the progression from these conditions toward mobility- disability and dementia, by disrupting attention, motivation, emotion, decision making, executive functions, motor control, learning and memory consolidation [1]. Micro-architectural sleep features are central to this link and a reduction in the cyclic alternating pattern (CAP), an electrophysiological marker of NREM sleep instability, is an example. Impaired sleep microarchitecture, as expressed by CAP impoverishment, reduces glymphatic clearance during sleep, increasing β-amyloid burden accumulated across the day (Fig. 1). It may function both as an early marker and a driver of neurodegeneration, identifying MCI patients at high risk for dementia and those with alpha-synucleinopathies such as Parkinson’s disease [2–7].

**Fig. 2:**
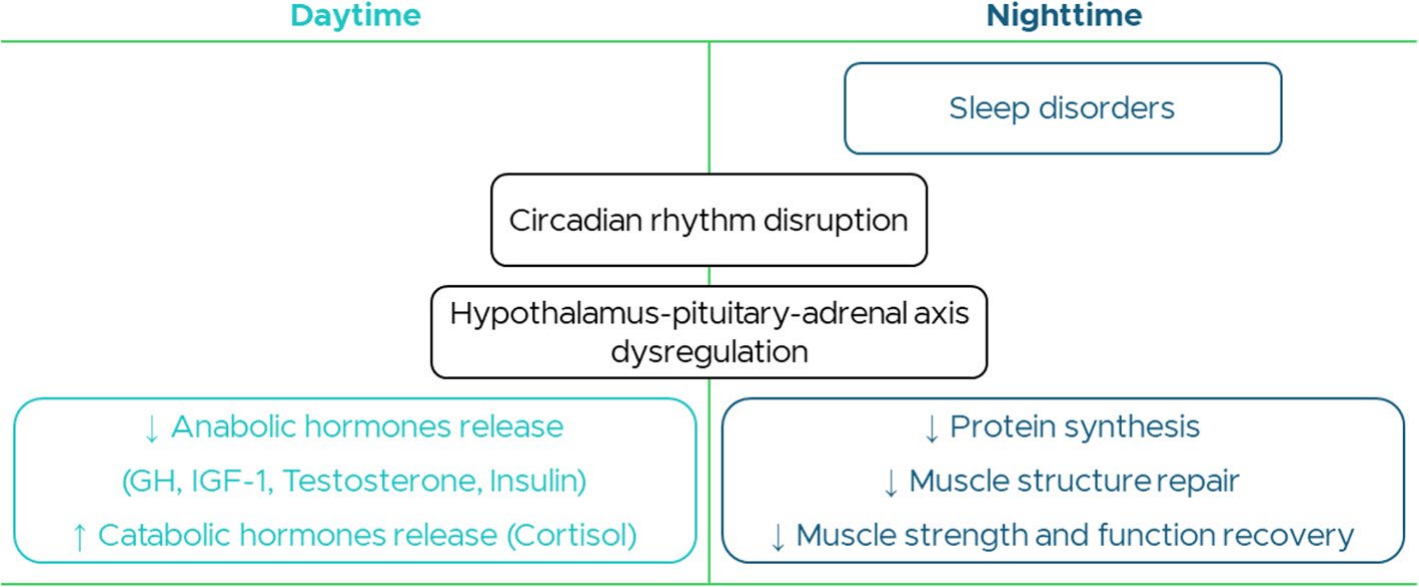
Schematic representation of the restorative theory

Sleep anomalies—including poor quality, extreme duration (short or long), prolonged latency, sleep-disordered breathing, and daytime sleepiness—associate with frailty [8]. Sarcopenia, a major determinant of physical frailty, is modulated by sleep via anabolic and catabolic hormonal pathways. In particular, protein synthesis, muscle repair, and the release of growth hormone (GH), insulin-like growth factor 1 (IGF-1), testosterone, insulin, and cortisol are sleep-dependent and circadian-modulated, as evidenced by the dysregulation of the hypothalamus–pituitary–adrenal axis (HPA) due to chronic insomnia [9] (Fig. 2).

Therefore, restoring favorable sleep patterns may physiologically re-activate somatotropic and HPA axis function, supporting muscle recovery and delaying age-related sarcopenia [10]. Observational data suggest a U-shaped relationship between sarcopenia risk and sleep duration: both short and long sleep, relative to normal duration, increase risk in community-dwelling older people [11]. Consistently, short (≤ 5 h/day) and long (≥ 8 h/day) sleepers show lower lean mass and higher fat mass; these sleep characteristics shape body composition and physical performances, being foundational to sarcopenic obesity [12]. This relationship between sleep disorders and sarcopenic obesity is bidirectional [13]. Specifically, EPISONO and ELSA-Brasil cohorts link obstructive sleep apnoea (OSA) and nocturnal hypoxia to sarcopenic obesity [14], while OSA risk is higher in older obese adults with low muscle mass and strength [15]. Hence, coexisting sleep disorders, sarcopenia, and adverse body composition potentiate each other’s consequences, underscoring intervention targets across this intertwined pathophysiology—often initiating in midlife [16].

Cardiovascular diseases (CVD) mediate the sleep–frailty nexus, through bidirectional sleep and cardiac-skeletal muscle interactions [17]. Evidence, including our past research experience [18], demonstrates frequent coexistence of CVD and frailty, with the latter worsening outcomes and recovery of the formers [19]. In older sarcopenic and physically frail adults, appendicular lean mass positively correlates with left ventricular mass, cardiac output [20], and systemic blood pressure—the main determinant of LV mass [21]. This linkage between myocardial structural changes and skeletal muscle status lays the ground for a cardiac–skeletal muscle axis well established in ‘cardio-sarcopenia’ and firstly studied in European older cohorts [22]. Thus, sleep-disordered breathing, insomnia, fragmentation, and deprivation impair autonomic control, increases oxidative stress and inflammation [23], and disrupts endothelial function, elevating risks of CVD (E.g., hypertension, heart failure, coronary disease, arrhythmias), metabolic disorders (I.e., diabetes and obesity), and frailty [24]. Therefore, early, comprehensive, and multidisciplinary detection of compromised sleep health is critical to reduce cardiometabolic burden and to mitigate frailty onset and progression [25]. Together with muscle and adipose tissue, bone is the third component of a triad that is fundamental to the physical performance of older individuals, as well as contributing factor for physical frailty. Observational and interventional studies suggest that short sleep duration and night shift work negatively impact bone metabolism and health through undefined mechanisms, involving sleep and circadian rhythm disruption, which seems to impact bone formation more than bone resorption [26].

### Lifestyle factors and medications affecting sleep health

Regular physical activity and appropriate eating habits, among the others sleep hygiene measures, including daylight exposure, consistent sleep schedules, smoking cessation, and avoidance of alcohol, caffeine, stress, and intense noise, offers low-cost population-level benefits [27].

The effects of exercise on sleep in older adults remain heterogeneous. Aging degrades sleep quality, increasing daytime fatigue, lowering physical activity and wellbeing, and raising risks of mental, cardiovascular, and metabolic illness [28]. Physically active older adults report fewer sleep complaints, improved sleep efficiency, longer duration, and less effort initiating sleep [29]. Moderate–vigorous activity associates with better sleep quality in small studies [30] and physical activity above 150 min/week may complement insomnia management [31]. Medical sleep monitoring instruments could increase the accuracy of both sleep quality assessment and physical activity evaluation, contributing to determine its real impact on sleep health in older adults [32].

Nutrition influences sleep and age-related disease trajectories [33]. Caloric intake, macro- and micronutrients, alkaloids, and alcohol modulate wakefulness and sleep quality [28]. Low protein intake correlates with poor sleep, whereas high protein consumption may impair sleep maintenance [34, 35], as well as tryptophan—a serotonin/melatonin precursor—supports performance and total sleep time in adults consuming tryptophan-rich foods [36, 37]. In addition, the quality of carbohydrates consumed appears to be crucial for a healthy sleep. Fiber content and processing alter glycaemic index and meal timing, affecting sleep via cortisol/GH/glucagon release and inflammatory pathways [38]. Diets high in sweets or irregular meals associate with poor sleep, whereas whole grains, fruits, vegetables, and seafood support healthy sleep [28]. Moreover, correcting B-complex and vitamin D deficiencies may improve sleep rate and quality [39, 40], as well as magnesium may reduce insomnia through NMDA antagonism, GABA agonism, and melatonin regulation [41]. Furthermore, the correlation between diet, gut microbiota, and the gut–brain axis provides such additional mechanisms as immune signalling, vagal modulation, blood–brain barrier and intestinal permeability, circulating microbial metabolites and reactive oxygen species, as well as serotonergic pathways bidirectionally related to sleep [42].

Psychoactive substances and drugs profoundly affect sleep. Caffeine impairs sleep onset/maintenance by prolonging sleep latency and reducing total duration and quality [43, 44]. Tobacco use associates with reduced sleep efficiency, REM suppression, and increased daytime sleepiness [45], through central nicotinic effects on acetylcholine, glutamatergic, dopaminergic, and serotonergic signalling [46]. Alcohol disturbs sleep architecture and duration, as well as circadian timing [47].

Medications may induce daytime sleepiness (antihistamines, anticholinergics, anticonvulsants, opioids) or insomnia (pseudoephedrine, β-agonists, corticosteroids, antidepressants, methylphenidate, selegiline) [48].

Drug-related exacerbation of comorbid conditions (e.g., respiratory decline or OSA with β-blockers, benzodiazepines, opioids; diuretic-induced polyuria; ACE-inhibitor–related cough) further disrupts sleep. Polypharmacy—commonly defined as ≥ 5 concurrent drugs—raises risks of drug-related problems and adverse outcomes in older adults [49]. Drug–drug and drug–disease interactions and prescribing cascades amplify harm to sleep health [50].

Individually or interacting with each other, these mechanisms can have exponentially deleterious effects on sleep health.

**Fig. 3:**
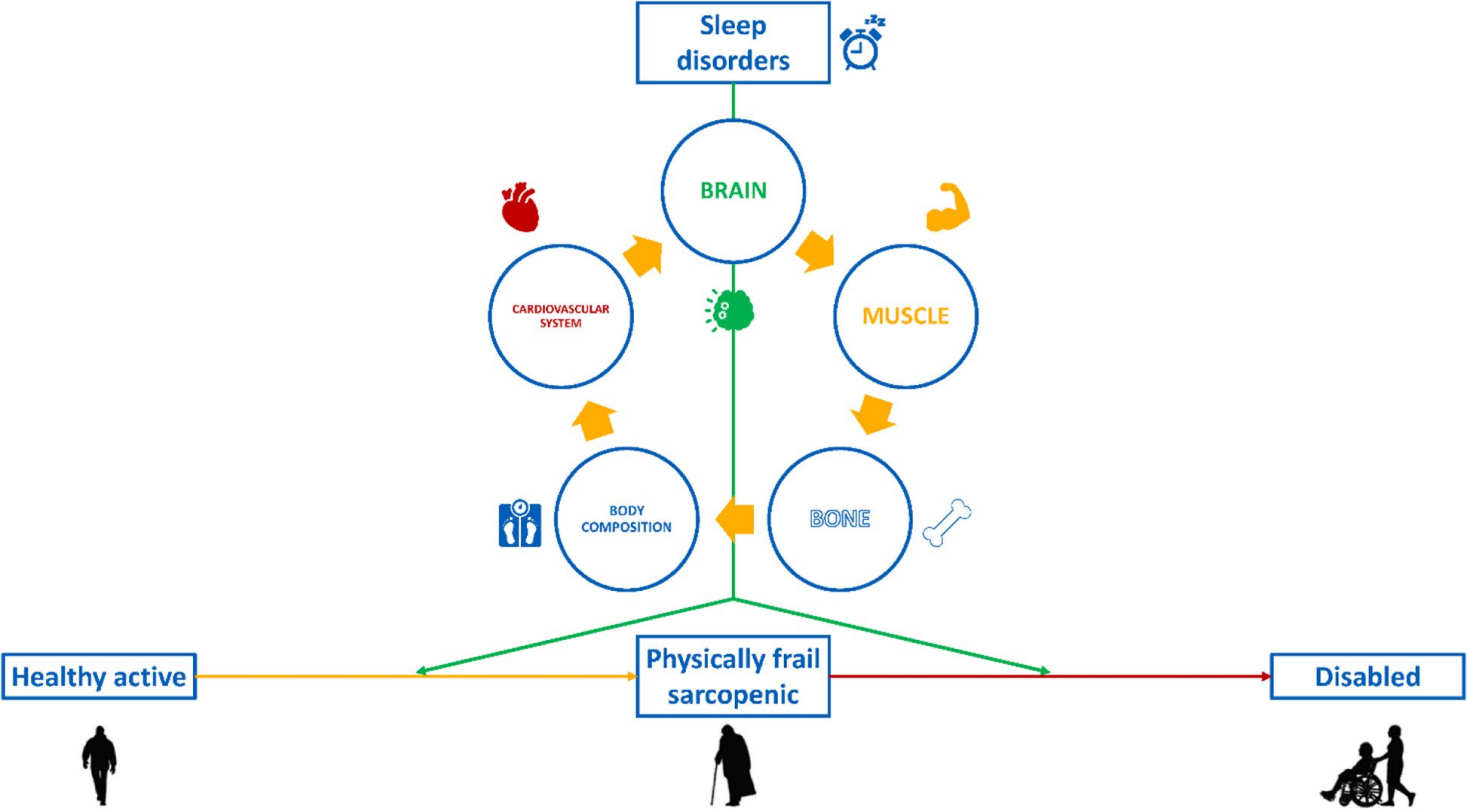
Impact of sleep disorders on ageing trajectories and related pathophysiological implications

The purpose of our work is to achieve a deeper understanding of how sleep and sleep disorders impact on mild cognitive impairment and cognitive frailty, sarcopenia and physical frailty, accelerating the trajectory toward dementia and mobility disability, leveraging advanced diagnostics and digital health techniques. The analysis will be focused on reciprocal relationships and carried out on both healthy active and physically frail sarcopenic elderly people. In particular, our objective is to detect the changes in brain, muscle, hormonal milieu, cardiovascular system, skeletal and body composition, by outlining their different intertwined trajectories in individuals with and without sleep disorders, in a comprehensive and translational framework (Fig. 3).

## Methods

### Objectives

Detect, in a comprehensive and translational way, changes in brain, muscle, hormonal milieu, cardiovascular system, skeletal and body composition to gain a deeper understanding of the impact of sleep disorders on mild cognitive impairment and cognitive frailty, sarcopenia and physical frailty, dementia and mobility disability.

The primary objective is to evaluate the effect of sleep disorders on the incidence of physical and functional worsening from the baseline to 12 months [51–53].

The secondary and exploratory objectives are listed in Table 1.

**Table 1:** List of secondary and exploratory objectives/outcomes

**Secondary objectives/outcomes** *To be evaluated in healthy active and/or physically frail sarcopenic older individuals with and without SD*
Prevalence and incidence of sarcopenia *according to sex specific cut-points recommended by EWGSOP2* [54]
Prevalence of MCI and cognitive frailty
Effect of sleep disorders on the incidence of MCI and cognitive frailty
Effect of sleep disorders on the incidence of dementia in cognitively frail participants
Prevalence and incidence of recognised pre-frailty conditions (I.e., MCR)
Prevalence and incidence of malnutrition, polypharmacy and multimorbidity
**Exploratory objectives/outcomes** *To be evaluated in healthy active and physically frail sarcopenic older individuals with and without SD*
Radiological, ultrasonographic, and bioimpedance changes in bone, muscle, and body composition
Magnetic resonance and nuclear medicine brain changes
Ultrasonographic and functional cardiovascular system changes
Anthropometric parameters and nutritional measures changes
Quality of life, social well-being, and reward level changes
Use of healthcare services changes
Differences in mortality
Criterion validity between MMSE score and brain MRI/Amyloid PET
Criterion validity between neuropsychological assessment and brain MRI/Amyloid PET
Role of recognised pre-frailty conditions (I.e., MCR) in affecting the adherence to a multi-component treatment

*Abbreviations*: *SD* Sleep disorders, *MCI* Mild cognitive impairment, *MCR* Motoric cognitive risk syndrome, *MMSE* Mini mental state examination, *MRI* Magnetic resonance imaging, *PET* Positron emission tomography

### Study design

**Fig. 4:**
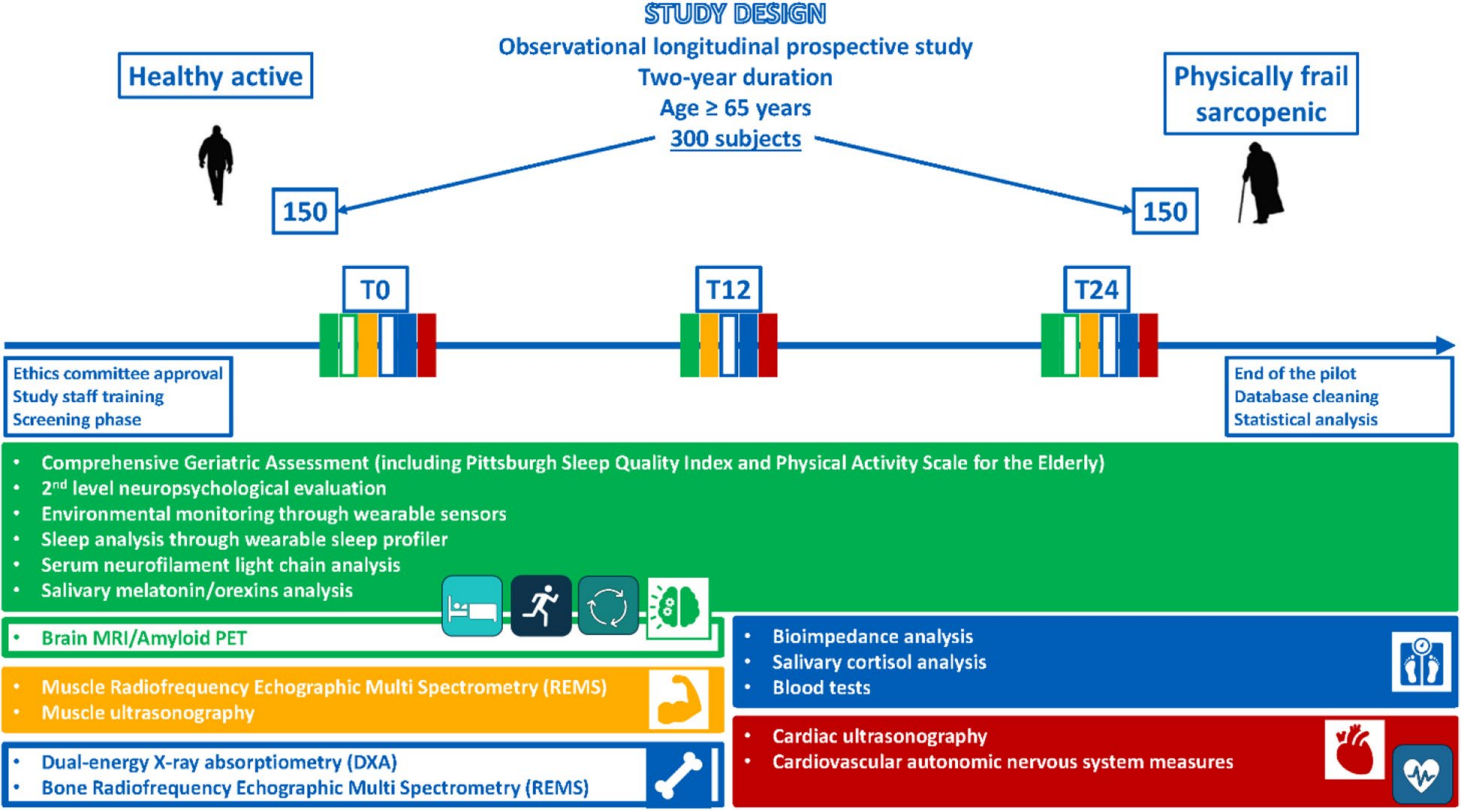
Schematic representation of the SOMNUS-DARE study design

The SOMNUS-DARE is prospective cohort study improving both the previous SPRINTT project [18] and the current Trajector-AGE (Clinical Trial Number: NCT06168591; Clinical Trial Registry: ClinicalTrials.gov; Registration Date: December 4th, 2023) study [55] expertise. The exposure is represented by the sleep disorders, and the primary outcome is the physical and functional worsening. The duration of the study and the follow up shall be nearly 24 months, according to the time of enrolment in the study and interim power reassessment. Upon approval by the Ethics Committee, the first 6 months will be dedicated to the team members’ training, the participants' enrolment, and the database setup and logistics arrangement. In order to maximize the statistical power of the analyses, each participant will be insured the same follow-up's length, according to the date of his/her recruitment. Those who will be enrolled at the beginning of the recruitment phase will have the same follow-up duration compared with those who will be included at the end of recruitment. Therefore, a 2-year follow-up will be guaranteed. Participants’ follow-up will take place at 3 time points: baseline, 12 and 24 months, by performing the measurements described below in the specific chapter (Fig. 4). The final 6 months of the study will be devoted to progressively carrying out of all the end-of-study visits and measurements, database cleaning and statistical analysis, as well as to predisposing both submission of papers to international scientific journals and disseminating results in international and national conferences.

### Setting and participants

Physically frail sarcopenic [3 ≥ Short Physical Performance Battery (SPPB) score ≤ 8] and healthy active [SPPB score > 8] [51] subjects from 65 years of age and afferent the geriatric and sleep medicine outpatient clinics, not demented and independent in the Basic Activities of Daily Living (ADL), will be categorized on the basis of the reduced sleep quality, self-reported and screened by the Pittsburgh Sleep Quality Index [PSQI (Italian version)] [56, 57] and stratified for the usual level of physical activity assessed by Physical Activity Scale for the Elderly [PASE-I (Italian version)] [58, 59]. The following eligibility criteria are intended to select a population that is at risk of accelerated decline in aging trajectory due to sleep disorders and their related systemic consequences. This age-group shall be selected for the sufficient long-life expectancy: to participate in a longitudinal study that lasts up to 2 years. Targeting this subset of the elderly population we will be able to recruit a non-disabled but at-risk population for a research project employing digital and eHealth technologies for frailty and disability assessment and prevention. Furthermore, we are planning to evaluate the eligibility criteria for those subjects previously enrolled in the SPRINTT clinical trial by the Parma site, completed at the end of 2019 and who have not evolved to a condition of disability or dementia, as well as those older individuals screened in the Trajector-AGE study by the same site, started at the end of 2022 and who were found to be sarcopenic and physically frail.

### Inclusion criteria

1. Age ≥ 65 years;
2. MMSE score [60] adjusted by age and education ≥ 25/30;
3. Absence of mobility-disability;
4. Absence of need for assistance with Basic Activities of Daily Living (according to the ICFSR International Clinical Practice Guidelines) [52].

### Exclusion criteria

1. Individuals with specific clinical conditions that may render the intervention unsafe (I.e., severe diseases, unstable health status);
2. Individuals whose adherence to the protocol might be low due to clinical (E.g., cognitive impairment, dialysis) and non-clinical (E.g., plans to relocate out of the study area within the next 3 years) reasons;
3. Individuals unable or unwilling to provide informed consent;
4. Consumption of more than 14 alcoholic drinks per week [one alcoholic drink (equal to 14.0 g of pure alcohol) corresponds to 36 cc of beer (5% alcohol content), 24 cc of malt liquor (7% alcohol content), 15 cc of wine (12% alcohol content), 4.5 cc of distilled spirit or liquor (40% alcohol content)];
5. Difficulty in communicating with the study staff due to speech, language, or (non-corrected) hearing problems;
6. Severe arthritis (E.g., awaiting joint replacement) that would interfere with the ability to participate fully in the study;
7. Lung disease requiring regular use of supplemental oxygen;
8. Severe cardiovascular disease (including New York Heart Association [NYHA] class III or IV, clinically significant congestive heart failure and valvular disease, history of cardiac arrest, presence of an implantable defibrillator or pacemaker, uncontrolled angina);
9. Upper and/or lower extremity amputation;
10. Peripheral arterial disease Lériche-Fontaine 3 or 4;
11. Renal disease requiring dialysis;
12. Current enrolment in another study involving lifestyle, nutrition, or pharmaceutical interventions;
13. Further medical, psychiatric, or behavioural factors that in the judgment of the principal investigator may interfere with the study participation;
14. Further possible illnesses affecting the life expectancy and reducing it to less than 24 months, corresponding to the study's length;
15. Clinical judgment concerning safety or non-compliance, as well as the conditions for which the use of the Sleep Profiler is not recommended (sensitivity of skin or scalp and/or open wounds on the forehead or scalp, allergic reactions to extended exposure to synthetic fabrics (e.g., polyester, rayon), upper respiratory infection or congestion, head circumference less than 21 or greater than 25 inches, forehead vertical measurement (from top of eyebrows to hairline) less than or equal to 2 inches or horizontal measurement (from hairline to hairline) less than or equal to 6 inches).
16. SPPB score < 3 at baseline.

### Withdrawal criteria

At any time, a participant could stop her/his participation in the study.

The reasons for early study's interruption will be:

1. Occurrence of any event that, according to the investigator, may interfere with the study participation or create an unjustified risk for the participant;
2. Illness or a condition leading to prolonged immobilisation;
3. Major deviation(s) to protocol incompatible with continuation in the study;
4. Any medical event requiring administration of an unauthorised concomitant treatment;
5. Non-medical reason;
6. Lost to follow-up: a patient can be declared by the investigator "lost to follow up", in case the investigator has no news about the participant, in spite of all the efforts to contact him/her, in order to find out the reasons for the interruption and convince him or her to come to the final visit.

### Data sources and measurements

**Fig. 5:**
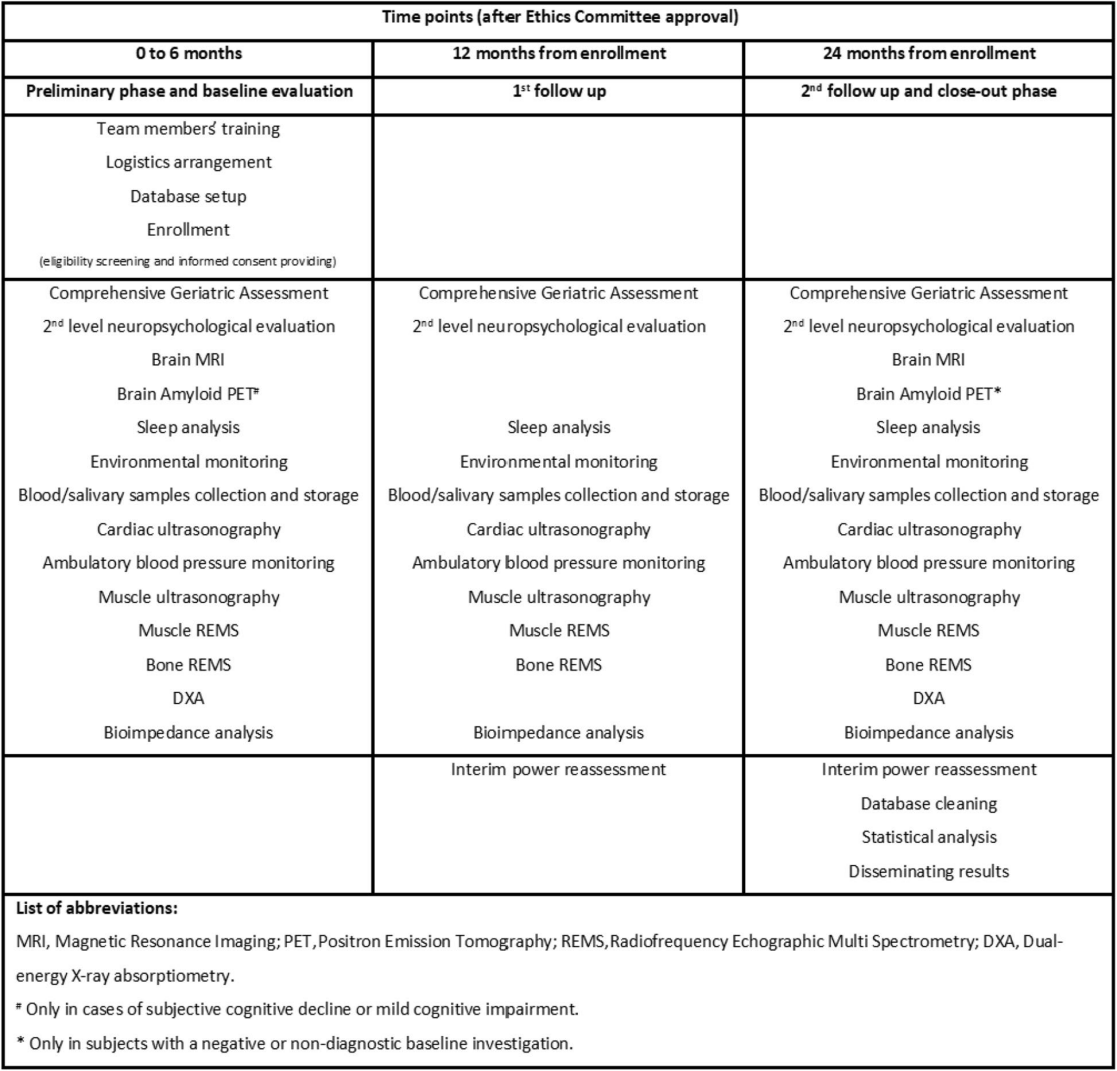
SOMNUS-DARE participant timeline

The tests time points are shown in the self-explanatory diagram below (Fig. 5).

Comprehensive Geriatric Assessment (CGA), including PSQI and PASE, 2nd level neuropsychological evaluation, provision of wearable devices for environmental monitoring and sleep analysis, as well as the training for their home use, collection of blood and salivary samples, muscle and bone Radiofrequency Echographic Multi Spectrometry (REMS), muscle ultrasonography and bioimpedance analysis (BIA) will be performed both at the Frail elderly lab of the Geriatric Clinic Unit and at the Geriatric Day Hospital, within the Interagency integrated activity department of continuity and multicomplexity of the University Hospital of Parma. The sleep profiling results analysis will be performed by the Sleep Medicine Centre, within the integrated activity department of general and specialty medicine. Brain MRI and Amyloid PET, Dual-energy X-ray absorptiometry (DXA) and cardiac ultrasonography will be performed within the interagency integrated activity diagnostic department and the Cardio-thoracic-vascular integrated activity department of the University Hospital of Parma, respectively. The remaining diagnostic tests and analyses (e.g., serum neurofilament light chain analysis, biomarkers of biological age, epigenetic clocks, inflammageing and muscle health related ones) will be performed either by specific labs within the department of Medicine and Surgery of the University of Parma or by external labs.

### Comprehensive geriatric assessment

A multidimensional panel of tools administered through a holistic clinical evaluation and aimed to analyse the bio-psycho-social sphere aspects listed below.

1. Cognition
  - Mini Mental State Examination (MMSE) [60] was originally proposed by Folstein and is widely used for the worldwide cognition assessment.
  - Clock Drawing Test is a first-order, rapid-fire test for the assessment of executive and visuospatial functions [61].
  - Trail Making Test (TMT, part A and B) is a test of visuo-motor speed (part A) and executive function (part B) [62]. In TMT-A, participants are asked to draw a line connecting circled numbers from 1 to 25 as fast and accurately as possible. The score corresponds to the time (0–300 s) required to connect the circles. In TMT-B, participants are asked to draw a line connecting ascending, alternating, numbers and letters (1-A-2-B-3-C, etc.). The score corresponds to the time (0–300 s) required to complete the task. The test is stopped if participants cannot complete it in 300 s or if five errors are made, in that case, the scores of 300 s and five errors are recorded.
  - Geriatric Depression Scale – 15 items (GDS) is a widely used and validated tool which show a significant agreement with the clinical diagnosis of depression [63].
2. Mobility and muscle performance
  - A 400-m walk test will be assessed using standardized conditions [64]. Participants will be asked to walk 400 m at their usual pace and without overexerting themselves. During testing, they may not use any assistive devices. Subjects will be instructed to begin the test at the start line from a standing position, to walk down the corridor. During the walk, if participants feel the need to stop and rest, they will be allowed to stand in one place and rest without sitting. They will be instructed to resume walking as soon as they will be able to do so. If they will be unable to continue after a 60-s rest stop or if they need to sit down, the test will be stopped. There will be no limits to the number of allowable rest stops, as long as the participant can complete the walk within 15 min without sitting. The test will be discontinued after 15 min, a time that corresponds to a very slow walking speed (0.45 m/s): translated into a walking capacity, it has little utility in daily life. Participants unable to complete the test within 15 min will be considered to be mobility disabled. Heart rate will be recorded at the beginning and at the end of the test. The number, timing, and reasons for the rest stops (fatigue, chest pain, feeling faint or dizzy, shortness of breath, or other) will be recorded. The inability to complete the 400-m walk has already been used as the defining criterion for mobility disability in major clinical studies, in particular the LIFE [65] and SPRINTT [18] trials.
  - Short Physical Performance Battery (SPPB) [51] was developed to objectively measure lower-extremity physical performance [66]. The test takes about 5 min to be administered. The battery has an excellent safety record. The components of the battery are the following:
    i. Standing balance, in which participants are asked to maintain balance in three positions, characterized by a progressive narrowing of the base support, with feet together (side by side position), the heel of one foot beside the big toe of the other foot (semi tandem position), and the heel of one foot in front of and touching the toes of the other foot (tandem position). For each of the three positions, participants have 10 s at longest. Scores are summed for the measure of balance for a range of 0 to 30 s.
    ii. Usual gait speed is assessed by asking the participants to walk at their usual pace over a 4-m course. Participants are instructed to stand with both feet touching the starting line and to start walking after a specific verbal command. Participants are allowed to use a cane if necessary, but not the assistance of another person or any other devices. The faster of two walks is used to compute walking speed.
    iii. Chair stands, in which the repeated chair stands test is performed using a straight-backed chair, whose back is placed against a wall. Participants are first asked to stand once from a sitting position without using their arms. If they are able to perform the task, they are then asked to stand up and sit down five times, as quickly as possible. The time to complete the task is recorded. To each of the three performance measures is assigned a score ranging from 0 to 4, with 4 indicating the highest level of performance and 0 the inability to complete the test. For the test of balance, participants are assigned a score of 1 if they can hold a side-by-side standing position for 10 s but they are unable to hold a semi-tandem position for 10 s; a score of 2 is assigned if they can hold a semi-tandem position for 10 s, but they are unable to hold a full-tandem position for 3 s; a score of 3 is assigned if they can stand in a full-tandem position for 3 s but less than 10 s; a score of 4 is assigned if they can stand in a full-tandem position for 10 s. Four categories are computed for walking speed and chair stands, according to standardized and previously published cut-points [67]. A summary score ranging from 0 (worst performers) to 12 (best performers) is calculated by adding walking speed, chair stands and balance scores.
  - Hand Grip Strength Test is a commonly used measure of upper-body skeletal muscle function and has been widely used as a general indicator of frailty with predictive validity for both mortality and functional limitation [68]. Grip strength is measured in the dominant hand using a hydraulic grip strength dynamometer: there are no known risks for the participant, except for a possible temporary discomfort during the test itself.
  - PASE is composed of 10 items: it requires 5 min, can be self-administered and has an easy scoring process. Furthermore, the activities evaluation, except for exercise, including the ones typical of older adults, such as household and caregiving, help to ensure that the instrument provides a comprehensive assessment of overall physical activity [58, 59].
3. Nutrition
  - 3-day dietary record through paper, pre-printed and blank will be delivered to participants. In these forms, participants will record the diet they followed during the 3 days before the clinical visit. In particular, for any food/beverage taken during the previous three days, participants will record time (date and hour), name of food/beverage, and quantity (portion, weight, volume).
  - Mini Nutritional Assessment – Short Form (MNA-SF) is a well-established instrument that allows the rapid assessment of nutritional status in elderly patients in outpatient clinics, hospitals, and nursing-homes. It has been translated into several languages and validated in many clinics around the world. The test is composed of simple measurements and brief questions that can be completed in about 5 min [69].
  - Global Leadership Initiative on Malnutrition (GLIM) criteria, a global consensus scheme for diagnosing malnutrition in adults in several clinical settings, will allow the diagnostic framing of malnourished individuals who have been identified as being at risk in the MNA-SF first screening step [70].
4. Multimorbidity
  - The Cumulative Illness Rating Scale (CIRS) will be used to measure the chronic medical illnesses burden, considering their severity stage [71].
5. Polypharmacy
  - Medication Inventory will allow to identify both prescription and non-prescription pharmaceutical products used by enrolled individuals. It is an important indicator of overall health, and the nature of the drugs taken is a strong indicator of clinically manifest disease. At each clinical visit all participants will be asked to bring all the prescription and non-prescription medications taken in the previous two weeks. The name, strength and formulation of each product are transcribed. These medications are coded according to the formulation for use in subsequent data analyses. This approach (drug assessment) has proved valid in older adults [72].
6. Functional status
  - The Lawton Instrumental Activities of Daily Living (IADL) score (ranging from 0 to 8; the higher the score, the greater the participant’s self-sufficiency) assesses the ability to perform tasks such as using a telephone, doing laundry, and handling finances. Measuring eight domains, it can be administered in 5 min. The scale may provide an early warning of functional decline or signal the need for further assessment [73].
  - Katz Activities of Daily Living (ADL) score (ranging from 0 if participant is totally dependent, to 6 if participant is independent) is used to collectively describe fundamental skills required to independently care for himself, such as eating, bathing, and mobility. It is used as an indicator of a person’s functional status and the inability to perform these activities results in the dependence from other individuals and/or mechanical devices, as well as may lead to unsafe conditions and poor quality of life. This score is a strong predictor of admission to nursing homes, need for alternative living arrangements, and hospitalization [74].
7. Quality of life
  - EuroQoL-5D-5L is a 5-item questionnaire exploring various domains of quality of life. In particular, subjects are asked to describe their current health status in terms of mobility capacity, self-care (e.g., washing, toileting, dressing), usual activities (e.g., work, study, hobbies), pain, anxiety or depression. Each of these domains is described by choosing one out of five possible degrees of impairment/severity. The EuroQoL-5D-5L score is calculated according to algorithms weighted on the European population, adjusting the value of the answers according to social and cultural influences of specific countries [75]. A negative EuroQoL-5D-5L score indicates a quality of life perceived as worse than death. The EuroQoL instrument also includes a 10-cm long visual analogue scale. Participants will thus be asked to draw a line from a box to the point on the thermometer-like scale (ranging from 0 – worst quality of life, to 100 – best quality of life) corresponding to their health-related quality of life.
8. Optimism
  - The Life Orientation Test-Revised (LOT-R) comprises 10 Likert-scaled items, with 6 assessing the main construct of dispositional optimism and the remaining 4 serving as filler items [76].
9. Sleep health
  - PSQI [56, 57] is a 19-item self-report questionnaire commonly used to measure sleep quality over the last month. Sleep quality, sleep latency, sleep duration, habitual sleep efficiency, sleep disturbances, use of sleeping medications, and daytime dysfunction are the assessed sleep health domains. They are scored as a global factor. If the global score is higher than 5, it is considered as an indicator of relevant sleep disturbances.
10. Other parameters
  - From baseline to the end of the study follow-up, at scheduled time points, participants will undergo assessment of vital signs (blood pressure, heart rate, and blood oxygen saturation) and main anthropometric measures (including body weight, calf circumference, waist circumference, hip circumference, and mid-arm circumference). Moreover, sociodemographic, economic and behavioural information for descriptive purposes (age, gender, race, living situation, household composition, marital status, educational level, smoking status, alcohol consumed, employment status, occupation, volunteer work, income level, and lifestyle habits) will be asked.

### 2nd level neuropsychological evaluation

During the neuropsychological evaluation, the neuropsychologist selects, administers, and interprets a battery of tests aimed to analyse cognitive major domains such as intelligence, attention/concentration, learning and memory, language, visuospatial and perceptual functions, executive functions, psychomotor speed, and sensory-motor functions. Tests are administered in a standardized manner and scores are interpreted by comparing the patient’s scores to an appropriate normative group. Furthermore, it involves a clinical interview, review of medical records, testing current cognitive and academic abilities, tests of social-emotional functioning and personality, adaptive functioning, behavioural observations, and integration of all these components [77].

### Brain MRI

Magnetic Resonance Imaging (MRI) on a 3 Tesla Magnetic field is the most detailed non-invasive medical imaging test to evaluate neuroanatomy at macroscopic, mesoscopic and microscopic levels [78] available in clinical practice.

Dysfunction of the glymphatic system has been implicated in several neurodegenerative diseases. Non-invasive in vivo imaging of the glymphatic system is expected to be useful in elucidating the pathophysiology of these diseases. Currently, MRI is the most commonly used technique to evaluate the glymphatic system in humans. Different imaging methods to study the glymphatic system will be included in the study: DTI-ALPS (diffusion tensor image analysis along the perivascular space); 3D-FLAIR (Fluid Attenuated Inversion Recovery) before and after gadolinium-based contrast agents (GBCAs) integrated with Contrast Enhanced Black Blood (CE-BB) sequences; rCBF (relative Cerebral Flow) derived from ASL (Arterial Spin Labeling) Perfusion [79].

In order to analyse brain changes occurring during the aging process and eventually related to sleep disorders, as well as to study the glymphatic system [80, 81]: this diagnostic test will be performed at both baseline and 24 months.

### Brain amyloid positron emission tomography (PET)

In view of the well-known interactions between sleep disorders, circadian rhythm disruption, decline in amyloid scavenging activity by the glymphatic system and the subsequent intensification of amyloid deposition, as well as their determinant role in the dementia continuum, especially for the preclinical phases, participants will undergo Amyloid PET at baseline (in cases of subjective cognitive decline or mild cognitive impairment, as in use in clinical practice) and, at 24 months of follow up, in those subjects with a negative or non-diagnostic baseline investigation. This nuclear medicine investigation is currently the test with the highest specificity for detecting cerebral amyloid deposition [82]. In addition, new and important correlations with brain MRI findings have been proved [83].

### Sleep analysis

The sleep macrostructure analysis will be carried out with the Sleep Profiler (Advanced Brain Monitoring, Carlsbad, CA, US), which will be worn by the participant at home for one night, after proper training. It will allow an automatic scoring of the sleep macrostructure parameters, which will be also supervised and manually corrected, by study collaborators with international expertise in Sleep Medicine within the University Hospital of Parma. The choice to use the Sleep Profiler is determined by the comparability between this device and the gold standard polysomnography, as well as by a better tolerability [84, 85]. It enables a configurable acquisition of up to six channels of electro-physiological signals, which sampling rate and gain are adjustable to acquire electroencephalographic (EEG), electromyographic (EMG), electrooculographic (EOG), electrocardiographic (ECG) and photoplethysmographic (PPG) signals. The device also measures sound via an acoustic microphone, while movement and position are measured via a 3D accelerometer.

To screen for sleep disorders suspected through the self-reported sleep quality (PSQI) and sleep macrostructure analysis (Sleep Profiler), the following in-depth diagnostic tests will be performed.

1. Standardized questionnaires
  - Epworth Scale (ESS) is a questionnaire consisting of 8 simple questions and allows an estimate of the severity of daytime sleepiness. A score higher than 10 is considered an index of pathological daytime sleepiness [86, 87].
  - Insomnia Severity Index (ISI) is a seven-item questionnaire for self-assessment of insomnia in the previous two weeks. It is a reliable and valid tool for identifying cases of insomnia and for estimating its severity. The overall score ranges from 0 to 28. Scores above 8 are considered pathological for insomnia [88, 89].
  - Morningness-Eveningness Questionnaire (MEQ) evaluates the predisposition toward circadian rhythm disturbances and consists of 19 questions. The overall score falls within a range of 16 to 86. A score less than or equal to 41 identifies the "serotonin" type, a score greater than or equal to 59 indicates the "morning" type, while a score between 42 and 58 indicates an "intermediate" type [90, 91].
  - REM-sleep behaviour screening questionnaire (RBD-SQ) measures the likelihood of suffering from a form of REM parasomnia called REM Behaviour Disorder (RBD). It is based on 10 items with affirmative or negative answer. As the score rises, the probability of suffering from RBD increases [92, 93].
  - Fatigue Severity Scale (FSS) [94, 95] is a nine-item questionnaire for self-assessment of fatigue level. Every item rates the severity of the individual's fatigue symptoms in the past week with a score from 1 to 7. Adding up the score of each individual item gives the total score. A total score of less than 36 suggests that you may not be suffering from fatigue. Scores above 36 are considered pathological for fatigue.
  - STOP-Bang questionnaire [96] evaluates the risk of OSA. It involves a series of eight yes-or-no questions which relate to known risk factors for OSA (Snoring, Tiredness, Observed apnoea, Pressure—Body Mass Index (BMI), Age, Neck circumference, Gender). The total number of affirmative answers together calculates the global score (0 to 2 for low risk, 3 to 4 for intermediate risk, 5 to 8 for high risk of obstructive sleep apnoea).
2. A Sleep Medicine specialist colloquium on sleep habits and symptoms, aided by a 7-days sleep diary, will enhance the diagnostic pathway for insomnia, circadian sleep–wake disorders, sleep-related breathing disorders, sleep-related movement disorders (RLS-PLMD) and REM-sleep parasomnias.
  - If RBD-SQ score will be greater than 5, the subject will undergo Video-Polysomnography, as recommended by current guidelines for sleep disorders diagnosis (International Classification of Sleep Disorders, III edition);
  - If ESS score will be greater than 10 and/or STOP-Bang score higher than or equal to 4, the subject will undergo one-night Cardio-Respiratory Recording;
  - If there will be a clinical suspect for periodic limb movement disorder, the subject will undergo Cardio-Respiratory Recording.
3. Video-Polysomnography (v-PSG) represents the gold-standard instrumental examination for recording human sleep [97] and is a multiparametric polygraphic examination which includes electroencephalographic channels recording of cortical electrical signals using surface electrodes. The examination is completely non-invasive and painless. It allows to recognise sleep stages and is fundamental for the diagnosis of some sleep disorders, such as REM parasomnias and REM-sleep behaviour disorder.
4. Cardio-Respiratory Recording (CRR) is a non-invasive examination which allows the measurement of various cardiological, respiratory and autonomic parameters during sleep [98]. After assembly (at the Center for Sleep Medicine) the subject will sleep at home. Gathered data evaluation allows confirmation and quantification of nocturnal respiratory events, as well as diagnosis of OSA. Disease severity is based on the amount of nocturnal respiratory events (Apnoea/Hypopnea Index, AHI). For mild OSA, AHI is between 5 and 15 phases/h; for moderate OSA, AHI is between 15 and 25 phases/h; for severe OSA, AHI is higher than 25 phases/h. The medical device (Nox T3s, Nox Medical USA, Alpharetta, GA, US) technically involves the application of:
  - thoraco-abdominal bands to detect respiratory movements;
  - pulse oximeters to monitor peripheral oxygen saturation trends;
  - nasal cannulas to monitor respiratory airflow;
  - position sensors to monitor positional changes during sleep;
  - plethysmographs to monitor peripheral pulse wave trends (an indirect index of blood pressure trends).

### Environmental monitoring

As part of our multiparametric evaluation, we will use two wearable devices from whose integrated monitoring we will be able to study physical activity, sleep and circadian rhythm.

The first one, called GENEActiv (Activinsight, Kimbolton, UK) [99, 100], is a wrist band medical device equipped with accelerometer, light exposure and skin temperature sensors. The second one is a medical device called Dynaport 7 (McRoberts B.V., The Hague, NL) [101, 102], which is positioned at the lower back through a waist band and fitted with accelerometer, gyroscope, barometer and temperature sensors.

The simultaneous application of these devices will take place for a duration of 7 days at the different time points, after proper training.

### Blood and salivary tests

Standard blood analysis will be performed at both baseline and every scheduled follow up time point. Blood samples will be sent to the diagnostic testing laboratories of the University Hospital of Parma for the assessment of the complete clinical biomarkers panel. Further and more specific laboratory investigations will also be carried out in other external diagnostic facilities.

Biological samples will be collected in the early morning, after an 8-h fast. An operator’s training will be performed defining the procedures for collecting, handling, storage, and shipment of biological specimens. The total amount of blood collected for standard blood work and the establishment of the biobank will be approximately 60 mL per visit. The local technician will immediately bring the biospecimens to the local laboratory where they will be handled and eventually prepared for storage from −20 °C to −80 °C, for future assessment of biomarkers in ancillary studies. Each tube will have a label reporting the unique ID code of the participant as well as the collection date. The laboratory will be in charge of labelling the tubes with the received ID. Biological samples will be stored at the department of Medicine and Surgery of the University of Parma. Due to the dynamic nature of the field of biomarkers related to “Geroscience” and Sleep Medicine, we decided not to pre-specify a list of biological variables to be studied. On the contrary, we identified some biological pathways (mentioned afterwards) of special interest for their relevance [103]. We plan to adopt state-of-the-art analytic approaches to examine these pathways when the biological samples will be analysed. These included biomarkers of biological age and sleep health are in particular:

1. epigenetic clocks (epigenomics, transcriptomics, proteomics, and metabolomics) and sleep related circulating microRNAs [104];
2. inflammageing (CXCL9, EOTAXIN, MIP-1 alpha, LEPTIN, IL-1beta, IL-5, INTERFERON alpha, IL-4) and inflammatory balance (TRAIL, INTERFERON beta, CXCL1, IL-2, TGF-alpha, PAI-1, LIF) biomarkers;
3. neuromuscular health related biomarkers (CPK, TNF, IL-6, Ferritin, Hsp72, Carbonylated Protein, P3NP and sTnT, C-terminal Fragment of Agrin and Neurofilament Light Chain);
4. salivary melatonin and cortisol levels;
5. blood and salivary orexins levels [105–107].

### Cardiac ultrasonography

In order to study the relationships between cardiac function, sarcopenia and physical frailty, already highlighted in our previous studies [20, 21], M-mode, two-dimensional, and Doppler Echocardiographic examination will be performed by an ultrasonography-experienced cardiologist of the University Hospital of Parma, using a commercially available, multihertz sector, 2 to 4 MHz probe equipped machine (Philips 5500G, Koninklijke Philips N.V., Eindhoven, NL; Samsung V8, Samsung Medison Co., Ltd., Seoul, KR).

### Ambulatory blood pressure monitoring

This diagnostic test will be performed according to the latest evidence, following an internationally recommended methodology [108] and using an apneABP instrument (Meditech Ltd., Budapest, Hungary). It will complete our analysis of cardiovascular system responses to sleep disorders in older people, especially those with known or new diagnosed hypertension.

### Muscle ultrasonography

This diagnostic test will be performed according to the latest evidence, following an internationally recommended methodology [109–111] and using a 22–2 MHz (1920 elements) linear array equipped machine (Philips 5500G, Koninklijke Philips N.V., Eindhoven, NL).

After 30 min of participant resting and lying in neutral supine position (head rest maximal inclination of 30°, arm loosely besides body, hips neutral, knees fully extended, feet in upright position), a B-mode vastus lateralis muscle ultrasound of the dominant side will be performed, in the same position and with the muscle relaxed. At 50% of the muscle length, with the greater trochanter and the proximal border of patella as proximal and distal landmarks respectively, the medial and lateral side of the muscle will be located and marked with a dermographic pencil. In the middle point of these marks and keeping the transducer probe in a longitudinal direction (in line with the muscle fiber fascicles), muscle thickness (from aponeurosis to aponeurosis), pennation angle (angle between the muscle fiber fascicles and the deep aponeurosis) and fascicle length will be measured. Turning the transducer probe 90° and using the extended field of view (EFOV) technique, cross-sectional area (both anatomical and physiological) and echo intensity will be measured, as well as muscle stiffness and muscle microcirculation will be evaluated through shear wave elastography and microvascular flow respectively. All these measurements will be repeated three times and the mean value of each one will be used. The total muscle length will be marked and reported, as well as muscle volume will be estimated through a specific formula. Finally, comparing anatomical and physiological cross-sectional areas at relaxed muscle state with the same parameters at contracted muscle state, muscle contraction potential will be evaluated (the mean of three repeated measurements of each type of cross-sectional area). Image J software will be used in post-production of images [112], as well as grey-level co-occurrence matrix (GLCM) for post-processing textural analyses.

### Muscle and bone radiofrequency echographic multi spectrometry

The REMS method (EchoS, Echolight S.p.a., Lecce, IT) has recently been developed and allows the quantification of bone mineral density by ultrasound analysis of bone cortical at the femoral and lumbar levels. Recent studies have shown that REMS has specificity and sensitivity values in the diagnosis of osteoporosis comparable to DXA [113, 114]. The advantage of the REMS method lies in both the absence of radiation and the rapid execution. This technique also allows to analyse the distribution of body components providing fat mass quantification and basal metabolism measurement during the same ultrasound scanning.

### Dual-energy X-ray absorptiometry

Total body fat mass and total bone-free lean mass will be acquired by total body scans using Dual-energy X-ray absorptiometry (DXA) using standardised protocols [115, 116]. Appendicular lean mass will be derived from the sum of lean mass in both upper and lower extremities.

### Bioimpedance analysis

Bioelectrical impedance analysis (BIA) is a method for measuring body composition, including muscle mass, body fat, and total body water. Alternating low and high-frequency electrical currents are sent through the water in the body via contact with electrodes to measure impedance. This is a well-known and tested method for body mass and muscular health assessment. Multi-frequency BIA (mfBIA) equipment (InBody S10, InBody Co., Ltd., Seoul, KR) makes it possible to assess a particular muscle as a whole, as well as looking at a muscle at the fiber level [117].

### Sample size

An overall sample size of 300 patients was defined according feasibility.

However, according with the primary endpoint and the study design, the statistical power estimation was performed.

More specifically, we simulated the width from the lower limit of 95% confidence intervals for the Ratio Between Two Proportions.

The following parameters and assumptions were included into the equation:

- overall sample size of 300 patients;
- assumption of the balanced numbers (1:1) of physically frail sarcopenic [3 ≥ SPPB score ≤ 8] and healthy active elderly [SPPB score > 8] individuals;
- assumption of the unbalanced numbers (2:1) of individuals with and without sleep disorders at the baseline;
- expected rate of 9% of SPPB score < 9 at 12 months in healthy active elderly individuals [SPPB score > 8] without sleep disorders [118–123];
- expected rate of 27% of inability to complete a 400 m walk test in less than 15 min (without sitting, stopping for more than one minute, receiving help, or using a walker) in physically frail sarcopenic elderly individuals [3 ≥ SPPB score ≤ 8] without sleep disorders [18];
- an overall risk ratio of 1.55 at 12 months for individuals with sleep disorders versus the ones without sleep disorders (pooled expected rate 18%);
- an alpha error of 5%;

Thus, to reject the null hypothesis the lower limits of the ratio must not be lower than 1.11 (corresponding to a 20% of the lower limit of the differences).

Participants will be enrolled at the Frail elderly lab of the Geriatric Clinic Unit within the Interagency integrated activity department of continuity and multicomplexity of the University Hospital of Parma, by acquisition of informed consent and if, upon the geriatric multidimensional assessment, the eligibility criteria given in this study protocol will be met.

### Data collection and management

Data collection will be carried out using the REDCap (Research Electronic Data Capture, Vanderbilt University, Nashville, TN, US) platform installed at the University Hospital of Parma, according to the operating instructions released by the Clinical and Epidemiological Research Unit of the University Hospital of Parma. A specific computerized data collection form (eCRF) will be prepared. The system will be accessible exclusively to authorized personnel specifically profiled (data entry operator, monitor, data analyst, etc.) by entering strictly personal credentials (user name and encrypted password). Quality of data will be guaranteed through automated controls active during the data entry process (pre-calculated fields, branching logic, self-validations) and during the data management procedures (deletion, display and filtering). A “Data Validation Program” will be defined in order to detail which data will be checked (validated) and to define the quality monitoring indicators aimed at verifying completeness (missing data) and the presence of deviations (non-adherence to the protocol). The system will also be able to record the log of accesses and all operations performed (Logging and Audit Trail). The eCRF will respect the principle of minimization, i.e. the inclusion of the collection of data strictly necessary for the purposes of the main and secondary objectives of the study, and will be made available for possible verification by designated representatives in the event of monitoring visits.

### Statistical methods

Data will be summarized using mean ± standard deviation (SD) or, for non-normally skewed distributions, as median and interquartile range (IQR). Incidence (mild cognitive impairment and cognitive frailty; dementia; recognised pre-frailty conditions; malnutrition; polypharmacy; multimorbidity), prevalence (mild cognitive impairment and cognitive frailty; recognised pre-frailty conditions; malnutrition; polypharmacy; multimorbidity) and mortality among healthy active and physically frail sarcopenic elderly individuals with and without sleep disorders will be compared using chi-square test, adjusted for age and sex.

According to the sample size estimation, 95% CIs will be calculated with Clopper-Pearson method and tested with a test for two proportions. For interpretative purposes multivariable logistic regression will be implemented. Moreover, to evaluate the outcome in terms of continue variable, multivariable ordinal logistic regression model will be used to estimate the association between the sleep disorders and the single unit of decrease in physical and functional score (SPPB score).

Since there will be multiple measures (baseline, + 12 months, + 24 months), the Mixed Model for Repeated Measure (MMRM) will be adopted for the longitudinal analyses.

The analyses will be centralized and performed by the Clinical and Epidemiological Research Unit at University Hospital of Parma using R-Cran and STATA statistical softwares.

## Discussion

Using some of the most advanced diagnostic and digital health techniques, we will carry out a translational and multidimensional analysis of the changes induced by sleep disorders at the cerebral, muscular, metabolic, hormonal, cardiovascular, skeletal and body composition levels, in order to gain a deeper understanding of their pathophysiological role in the genesis of physical frailty and other relevant geriatric syndromes, as well as in the progression of these conditions towards dementia and mobility disability.

A 2-year prospective cohort design could be well-suited to elucidate temporal relationships between sleep disorders and changes in physical and functional status among older adults, as supported by longitudinal studies demonstrating associations between them and multimorbidity, frailty, and cognitive decline. The rationale for this approach includes the ability to capture incident outcomes and dynamic changes in sleep patterns, which is critical given the bidirectional nature of the relationship between sleep and aging.

It is well-known that sleep, during aging, undergoes architectural changes and that sleep disorders have in older people, traditionally affected by chronic diseases, different epidemiological characteristics than in younger ones. However, the classification of sleep disorders in older people has remained unchanged.

The profile of the subjects enrolled in our study, defined by specific inclusion and exclusion criteria, was specifically tailored to minimise the effect of comorbidities on the genesis of sleep disorders. This approach was used to ensure that those participants identified in our population sample were affected more likely by sleep disorders of primary rather than secondary origin.

Strengths of this design include also the ability to adjust for confounders and generalize findings to community-dwelling older adults. Limitations include potential selection bias, reliance on self-report for some sleep parameters, and challenges in disentangling causality due to complex interactions between sleep, aging, and comorbidities. Despite these limitations, prospective cohort studies demonstrated to be a good standard for investigating the impact of sleep disorders on aging trajectories [124–127].

Our methodological choices, incorporating both subjective and objective measures, as recently recommended by the American Heart Association [128], could comprehensively assess sleep health dimensions and minimize measurement bias. Furthermore, age-adapted analytic procedures we are planning to adopt, could account for interindividual differences and age-related changes in sleep physiology, as highlighted by recent methodological reviews [126].

Anticipated challenges include participant attrition, adherence to sleep monitoring protocols, and potential confounding from comorbidities, psychosocial factors, and medication use. On the other hand, regular follow-up, use of user-friendly wearable devices, robust statistical adjustment for covariates, and optimized sampling frequency represent countermeasures we are planning to involve and which allow to capture sleep variability, and not to miss key biological relationships as well [129, 130].

The project will allow to analyse sleep disorders, a public health problem, in the older population, where it is extremely relevant condition not fully addressed. In particular, reliance on subjective sleep assessment through questionnaires in cognitively frail individuals is known to introduce reporting bias, which can lead to under- or overestimation of sleep disturbances. On the other hand, objective sleep assessment through devices like actigraphs, sleep profilers, and cardio-respiratory monitors can mitigate this bias by providing quantifiable sleep parameters. Therefore, the inclusion of both types of measures, in research and clinical protocols, is known to represent the best opportunity to ensure robust and clinically meaningful assessment of sleep in vulnerable populations, as each provides distinct information: subjective measures reflect perceived sleep quality, while objective measures correlate more closely with sleep architecture. This approach will allow us to assess the sleep misperception, a relevant aspect of sleep health in this population [131–133].

**Fig. 6:**
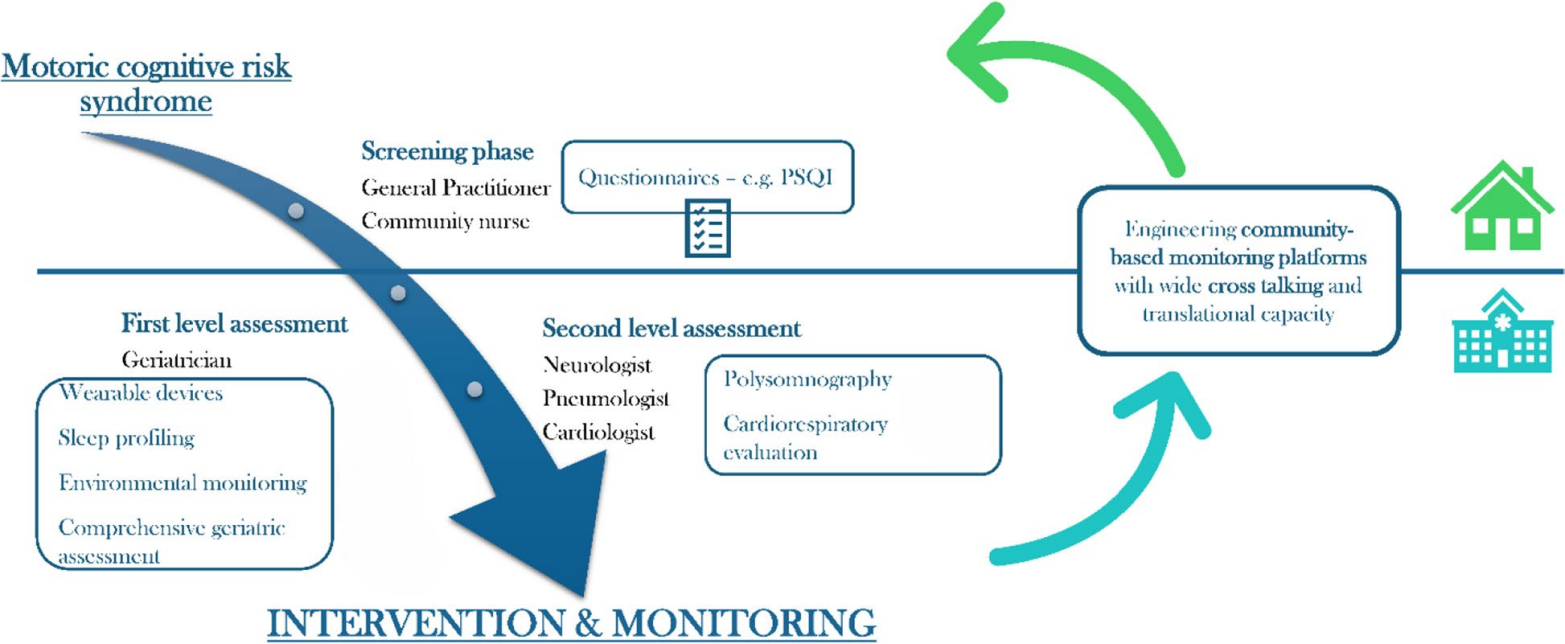
Multidisciplinary clinical pathway for the evaluation of sleep disorders in older people

By comparing the diagnostic capabilities of traditional techniques with the most innovative digital measurements in predicting the trajectories of physical, cognitive and quality-of-life functional decline, the expected results will highlight the importance of sleep as a fundamental determinant of ageing and healthspan. All these findings will allow the (digital health) improvement and personalisation of sleep disorders in a multidisciplinary assessment manner and to stimulate selective pharmacological treatment, and environmental hygiene measures (Fig. 6). These data will be translated in those individuals in whom routine therapeutic expedients have not provided substantial benefit.

### Outcomes

**Fig. 7:**
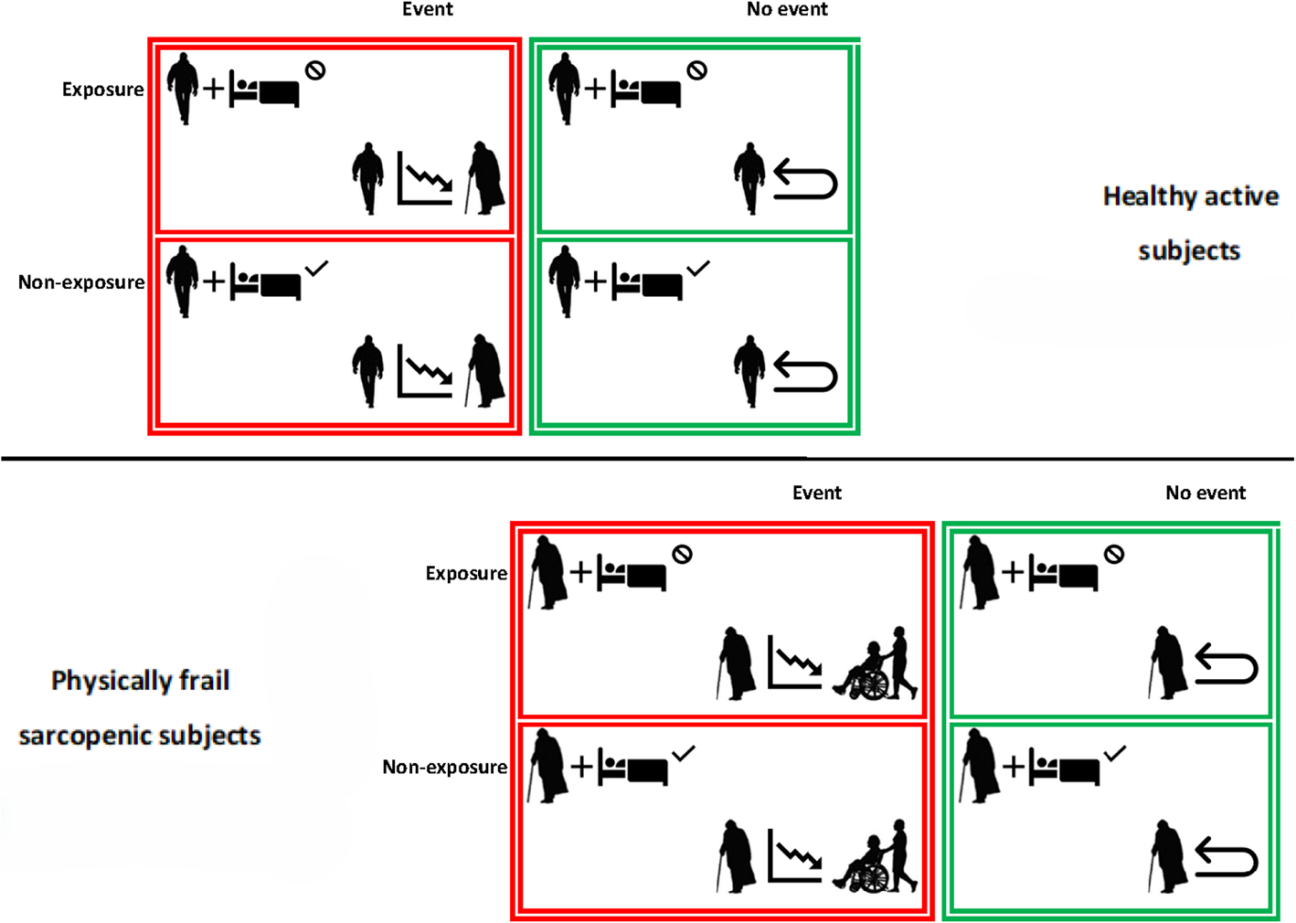
SOMNUS-DARE analytic aspects

The primary outcome is the incidence of physical and functional worsening at 12 months (Fig. 7).

It will be measured in terms of SPPB score < 9 [51, 52] for healthy active elderly individuals and in terms of inability to complete a 400 m walk test in less than 15 min (without sitting, stopping for more than one minute, receiving help, or using a walker) [53] for physically frail sarcopenic elderly Individuals.

The secondary and exploratory outcomes have already been listed in Table 1.

### Safety

All devices that will be used in the study are certified as medical devices.

All clinical tests that will be performed in the study are routinely used in the outpatient evaluation of older subjects.

All evaluations will be performed in the presence of a physician.

Only three minimally invasive measures, venous blood sampling, percutaneous infusion of PET radiopharmaceuticals and percutaneous infusion of MRI contrast, are included in the experimental protocol.

Any procedure that according to current legislation will require the acquisition of the subject's informed consent, will be performed only after the subject has been thoroughly informed about the risks and upon signed informed consent.

### Ethics and dissemination

This study will be conducted in accordance with the principles of Good Clinical Practice (GCP, Guideline for good clinical practice E6(R2), EMA/CHMP/ICH/135/1995). The Principal investigator agrees to adhere to the procedures and instructions contained therein and to carry out the study according to GCP Guidelines, the Declaration of Helsinki and the National Regulations governing clinical studies. Any changes to the protocol will be made in the form of an amendment. Any unexpected changes in the conduct of the study will be recorded in the "Clinical Study Report". The study protocol, any protocol amendment, informed consent and any other information for participants must be approved by the Ethical Committee. As far as the amendments are concerned, the Principal Investigator will be able to immediately apply them upon written communication to the Ethical Committee and without waiting for its approval, if the participating subjects’ safety will be at stake. Furthermore, if the Principal Investigator will believe that, for participants’ safety reasons, it will be necessary to immediately amend the protocol, he must notify the Ethical Committee within 10 working days. Each subject must provide written informed consent in order to participate in the study. The Principal Investigator will be responsible for archiving and storing the essential documents of the study, before, during the conduct and after the completion or interruption of the study itself, in accordance with the current legislation and the GCP Guidelines. All data collected will be strictly anonymous. All the subjects will be identified with a number. The Principal Investigator must retain the participants' original data and a copy of the signed written informed consent. Some data will be written directly on the CRF, which therefore will act as the original data. Inspections/verifications audits may take place to ensure that the study will be conducted in accordance with the protocol and applicable regulatory provisions (during its execution or after the study will be completed). If a Regulatory Authority will require an inspection, the Principal Investigator must immediately inform the Promoter and his Ethical Committee. The results of the study will be disclosed at the conclusion of the trial by means of publications in international scientific journals and abstract for national and international congresses. The study documents must be kept in a safe place to ensure confidentiality is maintained and may not be disclosed to others without written authorization, except to the extent necessary to obtain the subjects' consent to participate in the study. The Promoter will reserve the right to interrupt the study in respect of the participants' well-being. The telephone number and email of the contact persons for conducting the study will be reported in the Investigator Folder provided to the center.

## Data Availability

No datasets were generated or analysed during the current study.

## Abbreviation

MCI: Mild cognitive impairment
MMSE: Mini Mental State Examination
CAP: Cyclic alternating pattern
NREM: Non-rapid eye movement
OSA: Obstructive sleep apnoea
GI: Glycaemic index
NMDA: N-methyl-D-aspartate
GABA: Gamma-aminobutyric acid
REM: Rapid eye movement
ACE: Angiotensin-converting enzyme
EWGSOP2: European Working Group on Sarcopenia in Older People 2
MRI: Magnetic resonance imaging
PET: Positron emission tomography
SPPB: Short physical performance battery
ADL: Activities of daily living
PSQI: Pittsburgh Sleep Quality Index
PASE: Physical Activity Scale for the Elderly
ICFSR: International Conference on Frailty and Sarcopenia Research
NYHA: New York Heart Association
CGA: Comprehensive geriatric assessment
REMS: Radiofrequency echographic multi spectrometry
BIA: Bioimpedance analysis
DXA: Dual-energy x-ray absorptiometry
TMT: Trail making test
GDS: Geriatric Depression Scale
GLIM: Global Leadership Initiative on Malnutrition
MNA-SF: Mini Nutritional Assessment – Short Form
CIRS: Cumulative Illness Rating Scale
IADL: Instrumental activities of daily living
LOT-R: Life Orientation Test-Revised
DTI-ALPS: Diffusion tensor image analysis along the perivascular space
3D-FLAIR: Fluid attenuated inversion recovery
GBCAs: Gadolinium-based contrast agents
CE-BB: Contrast enhanced black blood
rCBF: Relative cerebral flow
ASL: Arterial spin labelling
EEG: Electroencephalographic
EMG: Electromyographic
EOG: Electrooculographic
ECG: Electrocardiographic
PPG: Photoplethysmographic
ESS: Epworth Scale
ISI: Insomnia Severity Index
MEQ: Morningness-Eveningness Questionnaire
RBD-SQ: REM-sleep behaviour screening questionnaire
RBD: REM behaviour disorder
FSS: Fatigue Severity Scale
STOP-Bang: Snoring, Tiredness, Observed apnoea, Pressure—Body mass index, Age, Neck circumference, Gender
BMI: Body Mass Index
RLS: Restless leg syndrome
PLMD: Periodic limb movement disorder
v-PSG: Video-polysomnography
CRR: Cardio-respiratory recording
AHI: Apnoea/hypopnea index
EFOV: Extended field of view
GLCM: Grey-level co-occurrence matrix
eCRF: Computerized data collection form
SD: Standard deviation
IQR: Interquartile range
MMRM: Mixed model for repeated measure
GCP: Good clinical practice
